# Optimization of Surfactant-Mediated Green Extraction of Phenolic Compounds from Grape Pomace Using Response Surface Methodology

**DOI:** 10.3390/ijms26052072

**Published:** 2025-02-27

**Authors:** Milica Atanacković Krstonošić, Darija Sazdanić, Mira Mikulić, Dejan Ćirin, Jovana Milutinov, Veljko Krstonošić

**Affiliations:** Department of Pharmacy, Faculty of Medicine, University of Novi Sad, Hajduk Veljkova 3, 21000 Novi Sad, Serbia; darijasazdanic@gmail.com (D.S.); mira.bursac@mf.uns.ac.rs (M.M.); dejan.cirin@mf.uns.ac.rs (D.Ć.); jovana.milutinov@mf.uns.ac.rs (J.M.); veljko.krstonosic@mf.uns.ac.rs (V.K.)

**Keywords:** Box–Behnken design, red grape pomace, surfactants, phenolics, extraction

## Abstract

Grape pomace is a by-product abundant in phenolic compounds that can be used in the food, cosmetic, and pharmaceutical industries. For the efficient extraction of such compounds, an aqueous solution of non-ionic surfactant Brij S20 was applied as a green extraction medium, and the optimization was performed using surface response methodology. The effects of four independent factors (surfactant concentration, time, pH, and solvent-to-material ratio) were evaluated, and total phenolic content (TPC), DPPH radical inhibition, and selected polyphenol compound concentrations were analyzed as responses. Using response surface methodology (RSM), five regression equations were derived and good adequacy of the models was confirmed. The solvent-to-material (SM) ratio was the most influential factor. Surfactant concentration of 3% (m/V), extraction time of 120 min, pH value of 4.06, and SM ratio of 50 mL/g were determined as optimum conditions to maximize all responses. Under the optimal conditions, the mean validated values obtained for TPC, DPPH, gallic acid, catechin, and quercetin concentrations were 968.50 ± 37.06 mg GAE/L, 61.41 ± 7.13%, 5.10 ± 0.05 mg/L, 10.62 ± 0.79 mg/L, and 6.04 ± 0.10 mg/L, respectively. Furthermore, the established conditions were applied for the extraction of phenolic compounds from grape pomace of four grape varieties. The proposed extraction method proved effective, providing extracts rich in polyphenols suitable for further applications.

## 1. Introduction

The grapevine (*Vitis vinfera* L., Vitaceae) is one of the most important cultivated plant species. It is grown on a large scale throughout the world and belongs to the world’s main agro-economic activities. According to the International Organization of Vine and Wine, 52% of total world grape production is destined for pressing, notably for the production of wine (47% of the total) and for the production of musts and juices. The remainder is intended for consumption as table grapes or dried fruit [1]. Millions of tons of residues are generated through viticulture and winemaking every year [2,3]. Grape pomace is a biodegradable solid by-product generated during wine production, representing about 25% of the weight of the grapes processed [4]. Grape pomace contains a significant amount of biologically active compounds, with phenolic compounds being the most abundant. It has been reported that grape pomace contains a broad spectrum of polyphenols, including flavonoids, anthocyanin, proanthocyanidins, and phenolic acids [5,6,7]. The biological potential of phenolic compounds of grapes, wine, and by-products has been studied in detail, and the most significant activities include antioxidant [8], anti-inflammatory [9], cardioprotective [10], anti-tumor [11,12], and neuroprotective [13]. Natural or even synthesized polyphenolics can reduce oxidative stress and chronic inflammation, key factors in the development of many pathological conditions. For example, isocoumarin analogs are used in order to develop target selective novel pharmacological entities and to reveal their mechanism of action as potential therapeutics [13,14].

One aspect of sustainability is repurposing or adding value to a certain by-product in order to reduce harmful environmental impact. Therefore, grape pomace, as a wine industry waste product, can be used as an inexpensive source of phytochemicals that may be applied in the cosmetics, pharmaceutical, and food industries [4].

Extraction is the first step in the recovery of polyphenols from plant-based materials [2,15,16]. In order to maximize the recovery of grape polyphenols from winery by-products with minimal energy and solvent consumption, as well as minimal degradation during the extraction process, the optimization of well-known conventional extraction techniques and the implementation of novel methods have become subjects of great interest [7,17,18,19]. Therefore, research into new alternatives to conventional solvents appears to be an important component in the development of environmentally friendly extraction technologies. The use of aqueous solutions of surfactants as solvents represents a new, green, and efficient approach to solid/liquid extraction [20,21,22,23]. Namely, non-ionic surfactant systems are of great interest due to their suitable chemical and physical properties [20,22]. To maximize surfactant-based extraction efficiency, extraction conditions (pH, surfactant concentration, SM ratio, temperature, extraction time, etc.) should be optimized [21,23]. In addition to the lengthy duration and scarce extraction efficiency, extraction methods of polyphenols with one variable at a time do not provide a clear insight into the existence of interaction effects among the variables and may lead to misinterpretation of results [24]. On the other hand, response surface methodology (RSM) is an effective statistical technique for the optimization of analytical parameters when one or more responses are affected by several factors and their interactions. It has been successfully used to optimize the extraction conditions of polyphenols using various extraction techniques from different materials [23,25,26,27,28,29]. However, to the best of our knowledge, a study on the optimal parameters for simple non-ionic surfactant-mediated extraction of phenolics from grape pomace has not yet been published. Therefore, the aim of this study is to apply response surface methodology to determine the optimal combination of independent extraction factors for the most efficient extraction of phenolic compounds. In our previous research, we tested different types of non/ionic surfactants (poloxamer, Brij, Triton, and Tween subgroups) as mediums for the extraction of phenolic compounds from grape pomace. The surfactant that was among the most efficient was Brij S20, providing extraction efficiency higher than water or ethanol as pure solvents [30]. Bearing in mind its availability and relatively low price, Brij S20 was chosen as the candidate for further optimization of the conditions for extraction of phenolic compounds from grape pomace using RSM. The varied factors were surfactant concentration, time, pH, and SM ratio. The analyzed responses included total phenolic content, the concentration of selected phenolic compounds (gallic acid, catechin, and quercetin), and DPPH radical inhibition. Furthermore, the aim is to apply the established optimal extraction conditions on grape pomaces from different grape varieties.

## 2. Results and Discussion

### 2.1. Response Surface Methodology Analysis

To identify the effects of process variables on the extraction of antioxidant phenolic compounds (total phenolic content, DPPH radical scavenging activity, concentrations of gallic acid, catechin, and quercetin) from grape pomace, the three-level Box–Behnken design was conducted using four variables (surfactant concentration, extraction time, pH, and SM ratio). Various combinations of uncoded experimental conditions with their respective experimental data (mean from three replicates) and the predicted values from the mathematical model are listed in Table 1.

### 2.2. Fitting of the Second-Order Polynomial Equation

Applying response surface methodology, the regression equations for the observed responses are expressed as follows:

Y_TPC_ (mg GAE/L extract) = 1170 + 74.7·*X*_1_ + 3.66·*X*_2_ + 4.0·*X*_3_ − 14.72·*X*_4_ − 5.40·*X*_1_*·X*_1_ − 0.00505·*X*_2_*·X*_2_ + 2.28·*X*_3_*·X*_3_ + 0.04548·*X*_4_*·X*_4_ − 0.101·*X*_1_*·X*_2_ − 1.03·*X*_1_*·X*_3_ + 0.0126·*X*_1_*·X*_4_ − 0.410·*X*_2_*·X*_3_ − 0.00241·*X*_2_*·X*_4_ + 0.0812·*X*_3_*·X*_4_ (1)

Y_DPPH radical inhibition_ (%) = 37.3 − 1.69·*X*_1_ + 0.012·*X*_2_ + 15.90·*X*_3_ − 0.473·*X*_4_ + 0.520·*X*_1_*·X*_1_ + 0.001185·*X*_2_*·X*_2_ − 1.407·*X*_3_*·X*_3_ + 0.001893·*X*_4_*·X*_4_ − 0.0132·*X*_1_*·X*_2_ + 0.207·*X*_1_*·X*_3_ − 0.0258·*X*_1_*·X*_4_ − 0.0187·*X*_2_*·X*_3_ − 0.000465·*X*_2_*·X*_4_ − 0.0146·*X*_3_*·X*_4_ (2)

Y_Gallic acid concentration_ (mg/L) = 3.28 − 0.083·*X*_1_ − 0.0262·*X*_2_ + 1.405·*X*_3_ − 0.0519·*X*_4_ − 0.0021·*X*_1_*·X*_1_ + 0.000226·*X*_2_*·X*_2_ − 0.1307·*X*_3_*·X*_3_ + 0.000216·*X*_4_*·X*_4_ − 0.00060·*X*_1_*·X*_2_ − 0.0006·*X*_1_*·X*_3_ + 0.00110·*X*_1_*·X*_4_ − 0.00051·*X*_2_*·X*_3_ − 0.000082·*X*_2_*·X*_4_ − 0.00172·*X*_3_*·X*_4_ (3)

Y_Catechin concentration_ (mg/L) = 13.39 − 0.265·*X*_1_ + 0.0050·*X*_2_ − 0.396·*X*_3_ − 0.1314·*X*_4_ − 0.0057·*X*_1_*·X*_1_ + 0.000045·*X*_2_*·X*_2_ + 0.0347·*X*_3_·*X*_3_ + 0.000499·*X*_4_*·X*_4_ + 0.00063·*X*_1_*·X*_2_ + 0.0245·*X*_1_*·X*_3_ + 0.00043·*X*_1_*·X*_4_ − 0.00140·*X*_2_*·X*_3_ − 0.000112·*X*_2_*·X*_4_ − 0.00013·*X*_3_*·X*_4_ (4)

Y_Quercetin concentration_ (mg/L) = 8.54 − 0.024·*X*_1_ + 0.0081·*X*_2_ − 0.018·*X*_3_ − 0.09605·*X*_4_ − 0.0113·*X*_1_*·X*_1_·+ 0.000014·*X*_2_*·X*_2_ + 0.0102·*X*_3_*·X*_3_ + 0.000307·*X*_4_*·X*_4_ + 0.00063·*X*_1_*·X*_2_ + 0.0048·*X*_1_*·X*_3_ + 0.001021·*X*_1_*·X*_4_ − 0.00168·*X*_2_*·X*_3_ − 0.000051·*X*_2_*·X*_4_ + 0.000566·*X*_3_*·X*_4_ (5)

### 2.3. Statistical Analysis and Estimation of the Adequacy of the Models

The ability of the obtained equations to describe the response’s variability was determined by analysis of variance (ANOVA), and the significance of the regression coefficients was evaluated by their corresponding *p*-values, as outlined in Table 2. The *p*-values for all models were less than 0.0001, indicating that all proposed models were statistically significant. The regression coefficients *X*_1_, *X*_3_, *X*_4_, *X*_1_*·X*_1_, *X*_4_*·X*_4_*,* and *X*_2*·*_*X*_3_ for TPC, *X*_3_ and *X*_4_, *X*_1_*·X*_1_, *X*_3_*·X*_3_, *X*_4_*·X*_4_*,* and *X*_1_*·X*_4_ for DPPH inhibition, *X*_3_, *X*_4_, *X*_3_*·X*_3_*,* and *X*_4_*·X*_4_ for gallic acid concentration, and linear *X*_4_ and quadratic *X*_4_*·X*_4_ for catechin and quercetin concentration were significant (*p* < 0.05). Since the interactive coefficients generally were not significant except *X*_2*·*_*X*_3_ for TPC and *X*_1_*·X*_4_ for DPPH inhibition, this indicates the presence of only a small interaction effect of the studied variables.

The lack-of-fit test for all models showed that there is no evidence that the model does not fit the data since *p*-values are larger than 0.05 (Table 2). Determination coefficients R^2^, adjusted R^2^, and predicted R^2^ were calculated for each model to verify the adequacy of the models to predict experimental data (Table 3). The values of R^2^ and adjusted R^2^ for all models are greater than 91% and 80%, respectively, which indicates the adequacy of the models, i.e., indicates that only a small percentage of the variation of experimental data is not explained by the respective models [31]. The values of the predicted R^2^ were higher than 93% for TPC, DPPH inhibition, and quercetin concentration, indicating a good model prediction of the responses for new observations, while predictive R^2^ of 54.81% and 75.64% for gallic acid and catechin, respectively, indicates lower predictive abilities of these models.

Residual plots were constructed in order to examine the adequacy of the models (Figure S1, Supplementary Material). Normal probability plots of residuals are used to verify the assumption that the residuals are normally distributed (Figure S1(AI–AV)). In the case of normally distributed residuals, that plot should follow a straight line. Since non-normal patterns are observed in normal probability plots of all models, the other residual plots are constructed to test the adequacy of the models. The residuals versus fits plots indicate that the residuals of all models are randomly distributed since they fall randomly on both sides of 0. A constant variance of residuals was observed for all models (Figure S1(BI–BV)). Moreover, on the residuals versus order plots (Figure S1(CI–CV)), no trends or patterns were observed, indicating that the residuals are independent of one another.

### 2.4. Effects of Process Variables

**Figure 1:**
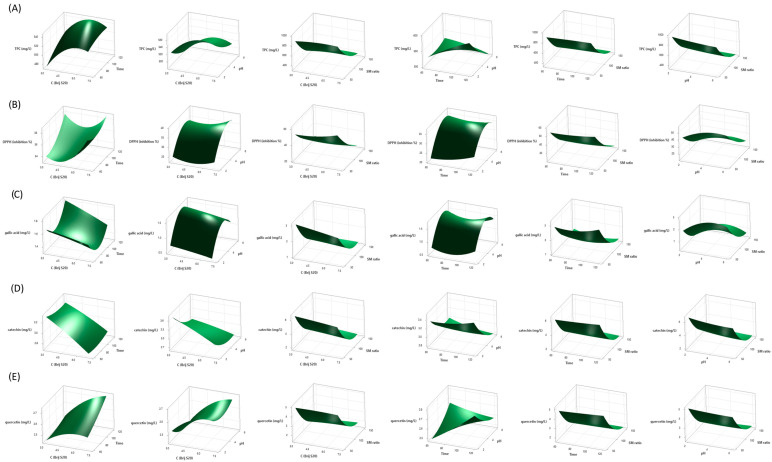
Response surface plots showing the interaction effect of process variables (C (Brij S20), time, pH, and SM ratio) on TPC (**A**), DPPH inhibition (**B**), gallic acid concentration (**C**), catechin concentration (**D**), and quercetin concentration (**E**). Only the interactive effect between C (Brij S20) and SM ratio was statistically significant (*p* = 0.0263) (Table 2). A study on optimization of the extraction of phenolic compounds from grape juice and wine pomaces using ethanol and acetone showed that four factors were significant for DPPH inhibition, including temperature, time, solvent type, and pomace type [33].

In order to show the relationship between the responses and experimental levels of each variable and to illustrate the interactive effects of independent variables (C (Brij S20), time, pH, and SM ratio) on the response variables (TPC, DPPH radical inhibition, and concentration of gallic acid, catechin, and quercetin) three-dimensional surface plots were generated using Equations (1)–(5) (Figure 1).

According to Figure 1A and the ANOVA results (Table 2), the concentration of surfactant, time, and pH had a positive linear effect on TPC (*p* = 0.0106, *p* = 0.0813, and *p* = 0.0024, respectively), while the SM ratio had a significantly negative linear effect (*p* < 0.0001). The most influential factor on TPC was the SM ratio in the linear model (percentage contribution of 88.60%). Quadratic terms of C (BrijS20) and SM ratio were statistically significant (*p* = 0.0273 and *p* < 0.0001, respectively), indicating curvilinear changes in TPC. The only significant interactive effect was the one between the time and pH (*p* = 0.0294). Namely, TPC increased and then slightly decreased with the increase in concentration of surfactant, and the highest TPC values were obtained around the midpoint. Accordingly, in recent research, the concentration of non-ionic surfactant Tween 80 positively affected the extraction of phenolic compounds from apple pomace, while the highest TPC values were observed around the midpoint [23]. A similar effect of concentration of non-ionic PEG 8000 on the extraction yield of polyphenols in single-factor experiments was observed. The total extraction yield of polyphenols increased when the concentration of surfactant increased from 0.05 mg/mL to 0.25 mg/mL, after which it slightly decreased. However, when the concentration of surfactant is excessive, lower extraction efficiency due to high viscosity and lower mass transfer between the matrix and solution can be expected [32]. As shown in Figure 1B, increasing the pH value and decreasing the SM ratio increased the DPPH radical scavenging activity of the obtained extracts. The linear and quadratic terms of pH value and SM ratio were statistically significant (*p* < 0.0001) (Table 2). The highest percentage contribution was observed for the SM ratio (77.54%). Regarding the influence of pH value, the highest DPPH inhibitory activity was observed in the midpoint at a pH of around 5 (Figure 1B). In Figure 1B, an increase in DPPH radical scavenging activity is observed with an increase in C (Brij S20) and time. The linear term of C (Brij S20) was negative, but the quadratic term was significantly positive (*p* = 0.0714 and *p* = 0.0353*,* respectively). The linear and quadratic terms of time were also positive, but both were statistically insignificant (*p* > 0.07).

Similarly to DPPH scavenging activity, the concentration of gallic acid in extracts increases with increased pH and decreased SM ratio, with these two linear terms being statistically significant (*p* = 0.0186 and *p* < 0.0001, respectively) (Figure 1C, Table 2). According to ANOVA results, the SM ratio was the most influential factor, with a percentage contribution of 54.81% (Table 2). Quadratic terms of pH value and SM ratio were also statistically significant (*p* = 0.0054 and *p* = 0.0042, respectively), explaining the curved response surfaces. Interestingly, it was noticed that C (Brij S20) had negative linear and negative quadratic effects on gallic acid concentration, even though both linear and quadratic terms were statistically insignificant (*p* = 0.3304 and *p* = 0.9563, respectively). On the other hand, the linear effect of time was negative, while the quadratic was positive but not statistically significant (*p* = 0.7152 and *p* = 0.2063, respectively). Interaction terms were not significant (Table 2).

According to ANOVA results, the efficiency of catechin extraction was most influenced by the SM ratio (percentage contribution of 75.66%), where the linear term of SM ratio and the quadratic term for SM ratio were the only statistically significant terms of the model (*p* < 0.0001) (Table 2). It was also observed that all linear terms except time, i.e., C (Brij S20), pH, and SM ratio, had negative effects on the concentration of catechin in the extracts (Figure 1D, Table 2).

The most influential factor in the concentration of quercetin in the extracts was the SM ratio (percentage contribution 87.10%) (Table 2). It can be observed that the SM ratio had a negative linear effect and positive quadratic effect on quercetin concentration, while these terms were the only statistically significant terms in this model (*p* < 0.0001) (Figure 1E, Table 2). Although statistically insignificant, linear terms such as C (Brij S20) and pH value had a negative effect on quercetin extraction, while time had a positive linear effect (Table 2).

Therefore, a decrease in the SM ratio led to enhanced TPC, DPPH inhibition, as well as the concentration of gallic acid, catechin, and quercetin, and the term of SM was the most influential and statistically significant in all models (Figure 1, Table 2). The SM ratio was the most influential factor in a simple surfactant-mediated extraction of phenolics from apple pomace when an aqueous solution of Tween 80 was applied as an extraction medium (independent variables in the experimental design were concentration range—0.1–1.9%, time—40–80 min, SM ratio—40–120 mL/g, and pH—2.0–6.0). In the mentioned study, an increase in concentration, time, pH, and SM ratio led to an increase in the extraction of phenolic compounds (mg GAE/g dry weight) until reaching certain midpoints. Namely, after reaching the SM ratio of around 100 mL/g, a slight decrease in TPC was observed [23]. In a microwave-assisted extraction of target polyphenols and furanocoumarins from fig leaves with aqueous solution of non-ionic surfactant PEG 8000 (0.25 mg/mL) as extraction medium, Box–Behnken experimental design revealed that an increase in the liquid/solid ratio from 15 mL/g led to a higher extraction yield of rutin (mg/g dry weight) until reaching the midpoint at around 20 mL/g, and then became lower [32]. Jeganathan et al. [34] observed that total phenolic content (mg GAE/100 g) in red grape extracts obtained by simple solid/liquid extraction with acid/ethanol solution decreases significantly with increasing solid/liquid ratio. In another study, the solid/liquid ratio caused a negative effect on the total phenolic content (g GAE/kg dry matter) of grape pomace extracts obtained by glycerol solutions in a homogenizer-assisted extraction [35]. However, when comparing these results, the way of expressing TPC and the range of the SM ratio must be taken into account. Although its influence can vary depending on the type of extraction, the SM ratio is generally recognized as one of the most influential factors in the extraction processes in different studies. It is also suggested that an increased SM ratio does not increase the total yield due to exceeding the minimum solvent required to dissolve all polyphenols [32]. Additionally, the negative effect of SM increase on polyphenol extraction could be a consequence of the limited amount of polyphenols present in the extraction material.

Time was generally the least influential factor (except for quercetin concentration, on which it had the second smallest impact) and positively affected all investigated responses except gallic acid concentration (Figure 1, Table 2). Similarly, extraction time was one of the factors with the least impact on the recovery of phenolic compounds (mg GAE/g dry grape pomace) from the pomace of Portuguese grape varieties using ethanol and acetone in the experimental design [36]. In a simple extraction of antioxidant phenolic compounds from grape juice and wine pomaces using ethanol and acetone, longer extraction time had a positive significant effect on DPPH inhibition %, while its effect on TPC (mg GAE/g) was also positive but not significant at level 0.05 [33]. The mass transfer of compound from matrix to solvent is related to the time. However, the mass transfer increases with time until the maximum extraction is achieved [33,37].

Furthermore, it is known that solution pH plays maybe even more important role in the efficient extraction of phenolic compounds. As shown in Figure 1A–E, TPC, catechin, and quercetin concentrations decrease with an increase in pH. The percent of DPPH radical inhibition and gallic acid concentration increased with an increase in pH, but maximum responses were observed in midpoints at a pH of around 5. Therefore, it can be concluded that extracts rich in antioxidant polyphenols could be obtained in the acidic region, which is in agreement with the literature data [20,23,38,39]. Namely, simple phenolic compounds are weakly acidic, and therefore, in acidic solutions, they are predominantly present in an undissociated state. Thus, when an aqueous solution of non-ionic surfactant is used as an extraction medium, the undissociated compounds possibly form stronger interactions with the hydrophobic micellar core, resulting in efficient extraction [20,23]. Acidic conditions are used for the extraction of polyphenols because they are generally more stable in low pH since hydroxyl groups remain non-ionized, preserving their antioxidant properties and preventing degradation reactions [40]. Additionally, ionizable species like polyphenols in uncharged form are more soluble due to the reduction of their intermolecular interaction [41]. Furthermore, some phenolic compounds (like gallic, caffeic, and chlorogenic acid) are not stable at high pH, and it is shown that these transformations are not reversible [42]. Similarly, medium pH value has an important influence on the degradation of flavonoids like quercetin. It is shown that flavonoids are prone to degradation in basic conditions and that a higher number of hydroxyl groups in a flavonoid molecule leads to their lower stability [43].

### 2.5. Selection of Optimal Conditions and Model Validation

Predictive models were used for the theoretical calculation of the optimal set of extraction conditions for obtaining extract with maximal TPC (mg GAE/L), DPPH radical inhibition (%), gallic acid, catechin, and quercetin concentrations (mg/L). The optimum conditions were determined to be a surfactant concentration of 3%, extraction time of 120 min, pH value of 4.06, and SM ratio of 50 mL/g. The predicted results under the optimal conditions obtained by RSM are presented in Table 4.

For validation of the models, triplicate experiments were performed under the optimized conditions. The mean validated values obtained for TPC, DPPH radical inhibition, gallic acid, catechin, and quercetin concentrations were 968.50 ± 37.06 GAE/L, 61.41 ± 7.13%, 5.10 ± 0.05 mg/L, 10.62 ± 0.79 mg/L, and 6.04 ± 0.10 mg/L, respectively. The experimentally obtained % of DPPH inhibition fell within the CI and PI of predicted values, while TPC value and concentrations of gallic acid, catechin, and quercetin determined in *Cabernet Franc* extract obtained under optimal extraction conditions were even higher than predicted. This indicates a satisfactory predictive ability of the mathematical model and the suitability for optimizing the extraction of polyphenols from grape pomace using aqueous solutions of non-ionic surfactants.

The application of water solutions of Brij S20 for the extraction of grape pomace polyphenols has several advantages. Preparation of these types of extracts does not require additional equipment or high electrical or thermal energy demands (like microwave-assisted or ultrasound extraction), making them appropriate for scaled-up production. Brij S20 is considered to be nature-friendly and biodegradable, and it is widely used in different types of pharmaceutical and cosmetic products [44]. Therefore, polyphenol extracts obtained with Brij S20 could be directly used for the preparation of these types of products. For example, emulsions could be suitable carriers for extracts rich in polyphenols. It has been shown that polyphenol extracts from different sources can be successfully incorporated into stable emulsions [45,46].

Additionally, surfactant-mediated extracts could facilitate the formulation of the final product due to their increased stability. For example, a stabilization effect was observed in surfactant Tween 20-based anthocyanin extracts of grape pomace [47]. Therefore, the “leave-in” surfactant in the obtained extract is not only a desirable ingredient of a potential final cosmetic product but also contributes to the overall stability of the extract. Non-ionic surfactants are also known to enhance polyphenol penetration in the skin and their inhibitory effects on certain enzymes involved in the process of senescence [48]. Moreover, surfactant recovery and reuse in methods such as cloud point extraction opens the possibility of developing even more sustainable and cost-effective methods in the future [49].

### 2.6. Analysis of Phenolic Content and Antioxidant Potential of Grape Pomace Extracts of Different Varieties

#### 2.6.1. Total Phenolic Content and DPPH Radical Inhibition of Grape Pomace Extracts of Different Varieties

After establishing the optimal conditions, the polyphenol content and antioxidant activity of grape pomace extracts from different varieties from Serbia (*Cabernet Franc*, *Cabernet Sauvignon*, *Merlot*, *Prokupac*, and *Probus*) were determined.

Values of TPC and DPPH radical inhibition in the obtained extracts are presented in Table 5. Total phenolic content values were in a range from 438.15 ± 2.73 mg/L to 968.50 ± 37.06 mg/L, depending on the variety. *Cabernet Franc* extract contained the highest total phenolic content, while the lowest TPC was in *Cabernet Sauvignon* extract. Pantelić et al. [50] investigated the total phenolic content (mg GAE/g) of extracts of the seeds, skin, and pulp of red grapevine varieties such as *Cabernet Franc*, *Cabernet Sauvignon*, *Prokupac*, *Merlot*, and others, obtained by methanol with 0.1% HCl. Grape seed and skin extracts of the *Prokupac* variety exerted higher total phenolic content compared to *Cabernet Franc*, *Cabernet Sauvignon*, and *Merlot*, while the pulp extract of *Merlot* had the highest TPC among these varieties. Interestingly, in the same work, seed and skin extracts of the *Cabernet Franc* variety exhibited the lowest TPC values, indicating that differences in the phenolic content of grapes could be due to various factors (such as seasonal variations of weather, climatic conditions, agronomical practices), in addition to the variety [50]. The antioxidant activity of the extracts of different grape varieties was consistent with the total phenolic content, with *Cabernet Franc* showing the highest DPPH radical inhibition, which is in correlation with the literature data indicating a high and significant correlation between the results of these two assays [51].

#### 2.6.2. Individual Phenolic Content of Grape Pomace Extracts of Different Varieties

A total of 9 individual phenolic compounds were detected and quantified in the obtained extracts (gallic acid, vanillic acid, catechin, rutin, quercetin, kaempferol, naringenin, hesperetin, and resveratrol) (Table 6).

Generally, the phenolic content in grape pomace extract varied depending on the grape variety. Catechin was the most abundant phenolic compound in *Cabernet Franc*, *Prokupac*, and *Merlot* grape pomace extracts, while in the pomace extracts of *Cabernet Sauvignon* and *Probus* varieties, rutin had the highest concentration. The highest concentrations of gallic acid and catechin were observed in grape pomace extracts of *Prokupac* and *Cabernet Franc* varieties, while *Cabernet Sauvignon* contained the lowest concentrations of these compounds. Flavan-3-ols, catechin, and epicatechin were the most abundant compounds in grape seed methanolic extracts of 25 clones of Serbian autochthonous grapevine variety *Prokupac* [52] and are generally known as compounds predominantly contained in seeds [53]. The grape seed extract of *Prokupac,* obtained by extraction with 0.1% HCl in methanol, contained the highest content of catechin compared to *Cabernet Franc*, *Cabernet Sauvignon*, and *Merlot* [50]. Additionally, in methanolic extracts of *Prokupac* grape seeds, a significant amount of gallic acid and its derivatives were detected compared to the amounts in other red grape varieties, such as *Black Burgundy*, *Gamay Noir*, *Muscat Hamburg*, and *Gamay Bojadiser* [54]. Pantelić et al. [50] also reported that grape seed extracts of *Cabernet Sauvignon* contained significantly higher amounts of catechin compared to *Cabernet Franc*. The lowest concentration of vanillic acid was present in the extract of *Cabernet Franc*, while the *Merlot* extract contained its highest content. *Cabernet Franc* pomace extract contained the highest concentrations of the flavonols quercetin and kaempferol among all examined varieties, while the lowest contents of these compounds were extracted from the *Prokupac* variety. On the other hand, *Cabernet Franc* extract contained the lowest concentration of the flavonol rutin, while it was the most abundantly present in *Cabernet Sauvignon* extract. Quercetin was the dominant flavonol only in *Cabernet Franc* extract, while in all other varieties, rutin was the most abundant flavonol. Flavonols generally were the dominant phenolic compounds found in methanolic extracts of grape skins of different red grapevine varieties such as *Cabernet Franc*, *Cabernet Sauvignon*, *Prokupac*, *Merlot*, etc. [50]. The same authors reported that the highest rutin concentration was observed in the *Cabernet Sauvignon* extract, which was in agreement with our results. Hesperetin was detected only in *Merlot* extract. All surfactant-rich extracts contained resveratrol, while *Probus* grape pomace contained the highest resveratrol concentration.

## 3. Materials and Methods

### 3.1. Reagents, Chemicals, and Solvents

The surfactant Brij S20 (polyoxyethylene (20) stearyl ether, Brij^®^ S20, Sigma-Aldrich, Darmstadt, Germany) was used for the extraction procedures in this work.

The following reagents were used in the analyses: Folin-Ciocalteu reagent (Mol, Stara Pazova, Serbia), DPPH (1,1-diphenyl-2-picrylhydrazyl) radical and crystalline monohydrate gallic acid from Alfa Aesar (Kandel, Germany), anhydrous sodium carbonate (Na_2_CO_3_) and sodium hydroxide (NaOH) obtained from Centrohem (Stara Pazova, Serbia), 35% hydrochloric acid (HCl, Lach-Ner, Neratovice, Czech Republic), ethanol 96% (Reahem, Novi Sad, Serbia), HPLC-grade acetonitrile and analytical grade dimethyl sulfoxide from J.T. Baker, Phillipsburg, NJ, USA). Distilled water (Institute of Pharmacy, Faculty of Medicine, Novi Sad, Serbia) was used for the preparation of the solutions.

Phenolic standards chlorogenic acid, p-hydroxybenzoic acid, vanillic acid, caffeic acid, syringic acid, p-coumaric acid, rutin, resveratrol, quercetin, *t*-cinnamic acid, naringenin, and kaempferol were purchased from Sigma-Aldrich (Darmstadt, Germany), while catechin and hesperetin were obtained from Fluka (Seelze, Germany). Gallic acid was purchased from Alfa Aesar (Kandel, Germany) and benzoic acid from Lach-Ner (Neratovice, Czech Republic). The purity of standards was up to 99%.

### 3.2. Grape Pomace Samples

The extraction material in this research was grape pomace from grapes (*Vitis vinifera* varieties *Cabernet Franc*, *Cabernet Sauvignon*, *Merlot*, *Prokupac*, and *Probus*) cultivated in Serbia and obtained after the production of red wine (controlled wild fermentation technique) in a local winery. The samples were subjected to lyophilization, ground, and stored in a freezer at −20 °C until the extraction procedures and analyses. All optimization experiments were conducted on the *Cabernet Franc* variety.

### 3.3. Apparatus and Instruments

The following instruments were used: Vortex Genie 2 mixer (Scientific Industries, New York, NY, USA), InoLab pH-meter (WTW, Weilheim, Germany), Centrifuge 2–5 (Sigma, Osterode am Harz, Germany), Hei-Standard magnetic stirrer (Heidolph Instruments, Schwabach, Germany), 8453 UV-Visible Spectroscopy System (Agilent, Santa Clara, CA, USA), analytical balance AS 60/C/2 (Radwag, Radom, Poland). In addition, an Agilent 1100 Series liquid chromatograph (Agilent, Santa Clara, CA, USA) equipped with a binary pump, an inline degasser, and a diode array detector (DAD) was used to identify and quantify certain phenolic compounds in the extracts.

### 3.4. Extraction Procedure

An aqueous solution of the non-ionic surfactant Brij S20 (3%, 5%, 7%, m/V) was used as an extraction medium. The desired pH value of the surfactant solution (2.00 ± 0.05, 4.00 ± 0.05, or 6.00 ± 0.05) was adjusted with 0.1 M HCl and 0.1 M NaOH. The extracts were prepared by accurately weighing the required mass of the ground sample and adding 10 mL of the extraction medium of appropriate concentration and pH in order to achieve a certain solvent-to-material ratio (50, 100, or 150 mL/g). The extraction was performed at room temperature under stirring with a magnetic stirrer at 300 rpm for predetermined time intervals (60, 90, or 120 min). The extracts obtained were subjected to centrifugation for 20 min at 3500 rpm. The resulting supernatants were filtered through membrane filters (0.45 µm, Agilent, Santa Clara, CA, USA) prior to further analyses.

### 3.5. Determination of Total Phenolic Content

Total phenolic content (TPC) was determined spectrophotometrically by the Folin–Ciocalteu method [55], using gallic acid as standard. Briefly, 1000 µL of Folin–Ciocalteu reagent solution (previously 10-fold diluted with distilled water) was added to 200 µL of properly diluted extract sample (10-fold dilution). After 6 min, 800 µL of Na_2_CO_3_ (7.5%, m/V) was added, and the mixture was stored for 2 h in the dark at room temperature. The absorbance was measured at 740 nm. The calibration curve of standard gallic acid (10–100 mg/L) was used to calculate the TPC. The results were expressed as mg of gallic acid equivalents per liter of extract (mg GAE/L extract)).

### 3.6. DPPH Radical Scavenging Activity

The radical scavenging activity of properly diluted grape pomace extracts (20-fold dilution) toward DPPH radicals was assessed spectrophotometrically at 515 nm against a blank, according to the procedure described previously [56]. Namely, 100 µL of diluted extract was mixed with 1 mL of freshly prepared DPPH radical in ethanol, and the reaction solution was adjusted with ethanol up to a final volume of 4 mL. A control sample was prepared by mixing DPPH solution and ethanol. After incubation in the dark at room temperature for 1 h, the absorbance was measured. The inhibition percentage of DPPH radical was calculated using Equation (6).

(6) $$\mathrm{DPPH}\;\mathrm{radical}\;\mathrm{inhibition}\;(\%)=\frac{A_{control}-A_{sample}}{A_{control}}\times 100$$

### 3.7. HPLC Analysis

The content of individual phenolic compounds in the extracts was determined by means of the HPLC method [57]. Chromatographic separation was carried out on a Poroshell 120 EC-C18 (4.6 mm × 100 mm, 2.7 μm) column. The mobile phase consisted of solvent A (distilled water with 0.1% glacial acetic acid) and solvent B (acetonitrile with 0.1% glacial acetic acid) with a gradient elution program. The gradient was as follows: 0–3.25 min, 8–10%B; 3.25–8 min, 10–12%B; 8–15, 12–25%B; 15–15.8 min, 25–30%B; 15.8–25 min, 30–90%B; 25–25.4 min, 90–100%B; and 25.4–30 min, 100%B. The total duration of the analysis was 30 min. The flow rate was 1.0 mL/min, the injection volume 10 μL, and the temperature was constant at 25 ◦C. Quantification was performed by the external standard calibration. The standard stock solutions (1–25 mg/L) were made using dimethyl sulfoxide as solvent. Detection wavelengths were set at 225, 280, 305, 330, and 360 nm to simultaneously monitor the selected phenolic compounds.

### 3.8. Experimental Design

Response surface methodology was employed to find out the optimum conditions for the extraction of phenolic compounds from red grape pomace. For the optimization experiments, grape pomace of the *Cabernet Franc* variety was used. A 4-factor, 3-level Box–Behnken design (BBD) was applied to evaluate the effects of the combinations of four independent variables (mass concentration of Brij S20 surfactant (*X*_1_), the time of the extraction procedure (*X*_2_), pH value of extraction medium (*X*_3_), and the SM ratio (*X*_4_)) on the antioxidant capacity and the extraction of total and selected individual phenolic compounds from grape pomace. The independent variables and their levels of BBD are given in Table 7. The type of surfactant and levels of independent variables were chosen based on our previous research [30] and literature data [23]. The response variables were TPC (mg GAE/L extract), DPPH radical inhibition (%), and the concentration of gallic acid, catechin, and quercetin (mg/L).

Regression analysis was performed based on experimental data from BBD and fitted to a second-order polynomial model (Equation (7)):

(7) $$Y=\beta_{0}+\sum_{i=1}^{k}\beta_{i}X_{i}+\sum_{i=1}^{k}\beta_{ii}X_{ii}+\sum_{i=1}^{k}\sum_{j=i+1}^{k}\beta_{ij}X_{ij}$$

where *Y* is response, i.e., TPC, DPPH radical inhibition, concentrations of gallic acid, catechin, and quercetin; *X_i_* and *X_j_* represent coded independent variables; *β*_0_ denotes the model intercept; *β_i_*, *β_ii_*, *β_ij_* indicate the coefficients of the linear, quadratic, and interactive effect, respectively; and *k* is equal to the number of the tested factors (*k* = 4 in this study).

### 3.9. Validation of the Optimized Conditions

In order to verify the validity of the statistical experimental strategies, triplicate experiments were performed under optimal conditions. The average value of the experimental data was compared with the predicted values of the optimized conditions.

### 3.10. Statistical Analysis

All experiments were carried out in triplicates, and the results were reported as mean ± standard deviation (SD). Box–Behnken experimental design and data processing were performed using the response surface methodology within Minitab 21.1.0. software (Minitab Inc., State College, PA, USA). The fitting capacity of the models was evaluated by analysis of variance (ANOVA). The significance of each term was taken into account at *p* ≤ 0.05.

## 4. Conclusions

In the present study, a green and efficient extraction method using an aqueous solution of non-ionic surfactant Brij S20 as an extraction medium was established for the recovery of phenolic compounds from red grape pomace. The effects of surfactant concentration, extraction time, solution pH, and SM ratio on total phenolic content, DPPH radical inhibition, gallic acid, catechin, and quercetin concentration of extracts were determined by response surface methodology. The results demonstrated that the models were statistically significant, and the SM ratio was the most influential and statistically significant factor in all developed models. Solution pH emerged as another important factor in the efficient extraction of phenolics by the proposed method. Extraction conditions were optimized, and the experimentally validated results closely aligned with the predicted values, confirming the model’s predictive accuracy. The optimal conditions were further applied for the extraction of phenolic compounds from grape pomace of different grape varieties (*Cabernet Franc*, *Cabernet Sauvignon*, *Merlot*, *Prokupac,* and *Probus*), the obtained extracts were characterized, and notable variations in phenolic content across different grape pomace varieties were observed. Therefore, it was shown that the use of simple micelle-mediated green extraction technology allows obtaining extracts with high concentrations of antioxidant phenolic compounds from grape pomace and could be used for further development of relevant products.

## Figures and Tables

**Table 1:** The Box–Behnken design with experimental and predicted data for the extraction of bioactive compounds from *Cabernet Franc* grape pomace.

Run	C (Brij S20) ^1^, % (*X*_1_)	Time, min (*X*_2_)	pH (*X*_3_)	SM Ratio ^2^, mL/g (*X*_4_)	TPC ^3^ (mg GAE/L Extract)		DPPH Radical Inhibition (%)		Gallic Acid Concentration (mg/L)		Catechin Concentration (mg/L)		Quercetin Concentration (mg/L)	
					Measured	Predicted	Measured	Predicted	Measured	Predicted	Measured	Predicted	Measured	Predicted
1	3	60	4	100	458.80 ± 0.84 ^4^	474.26	34.04 ± 1.14	33.93	1.45 ± 0.05	1.66	3.32 ± 0.04	3.30	2.47 ± 0.02	2.45
2	7	60	4	100	507.28 ± 0.29	521.55	37.22 ± 1.7	37.83	1.29 ± 0.07	1.53	2.78 ± 0.09	2.73	2.59 ± 0.01	2.54
3	3	120	4	100	511.79 ± 0.07	508.21	39.06 ± 0.92	37.80	1.76 ± 0.1	1.81	3.32 ± 0.11	3.20	2.66 ± 0.05	2.49
4	7	120	4	100	536.04 ± 1.19	531.27	39.07 ± 1.2	38.54	1.45 ± 0.09	1.53	2.94 ± 0.1	2.77	2.93 ± 0.02	2.73
5	5	90	2	50	971.82 ± 0.33	962.16	40.59 ± 0.87	42.61	1.48 ± 0.07	1.88	6.88 ± 0.08	6.63	5.32 ± 0.07	5.06
6	5	90	6	50	899.67 ± 0.66	899.12	55.86 ± 1.61	55.68	2.43 ± 0.09	2.78	6.40 ± 0.05	6.12	5.06 ± 0.08	4.92
7	5	90	2	150	388.95 ± 2.42	400.20	13.66 ± 1.04	13.20	0.53 ± 0.05	0.47	2.54 ± 0.14	2.64	1.83 ± 0.02	1.75
8	5	90	6	150	349.28 ± 1.5	369.63	23.10 ± 1.11	20.45	0.80 ± 0.07	0.68	2.01 ± 0.09	2.08	1.80 ± 0.02	1.83
9	3	90	4	50	868.79 ± 1.10	883.60	53.97 ± 2.13	53.12	2.97 ± 0.1	3.05	6.00 ± 0.11	6.50	4.70 ± 0.05	4.92
10	7	90	4	50	915.93 ± 1.16	916.26	61.36 ± 1.01	60.59	2.53 ± 0.12	2.63	5.45 ± 0.1	5.92	4.67 ± 0.03	4.89
11	3	90	4	150	337.40 ± 0.47	335.36	24.54 ± 1.01	25.95	0.99 ± 0.07	1.09	2.15 ± 0.08	2.41	1.61 ± 0.01	1.52
12	7	90	4	150	389.57 ± 0.34	373.05	21.62 ± 0.74	23.12	0.99 ± 0.09	1.10	1.77 ± 0.05	2.00	1.98 ± 0.05	1.89
13	5	60	2	100	537.22 ± 0.94	527.42	20.67 ± 0.55	21.97	0.70 ± 0.02	0.77	3.16 ± 0.15	3.36	2.44 ± 0.07	2.49
14	5	120	2	100	601.35 ± 1.37	598.45	27.24 ± 0.92	26.51	0.86 ± 0.05	0.91	3.13 ± 0.07	3.50	2.73 ± 0.04	2.81
15	5	60	6	100	528.63 ± 1.80	529.81	33.01 ± 1.05	34.38	1.24 ± 0.05	1.39	2.63 ± 0.01	2.99	2.61 ± 0.06	2.66
16	5	120	6	100	494.36 ± 0.07	502.45	35.08 ± 2.00	34.42	1.28 ± 0.11	1.40	2.27 ± 0.01	2.79	2.50 ± 0.03	2.58
17	3	90	2	100	526.45 ± 1.71	524.17	25.58 ± 1.16	24.92	0.94 ± 0.7	0.73	3.96 ± 0.03	3.71	2.44 ± 0.03	2.53
18	7	90	2	100	554.20 ± 1.90	567.59	27.06 ± 1.70	25.58	0.79 ± 0.02	0.53	3.20 ± 0.09	3.02	2.53 ± 0.01	2.66
19	3	90	6	100	507.98 ± 0.44	485.61	31.96 ± 0.93	33.43	1.52 ± 0.09	1.29	3.35 ± 0.1	2.98	2.50 ± 0.01	2.46
20	7	90	6	100	519.24 ± 0.93	512.54	36.75 ± 0.56	37.40	1.36 ± 0.07	1.08	2.99 ± 0.07	2.68	2.67 ± 0.07	2.67
21	5	60	4	50	910.98 ± 1.54	902.45	55.69 ± 1.5	53.99	3.45 ± 0.13	2.89	6.32 ± 0.11	6.12	4.91 ± 0.05	4.83
22	5	120	4	50	927.91 ± 1.43	931.51	56.20 ± 1.61	57.68	3.59 ± 0.08	3.21	6.69 ± 0.08	6.43	5.07 ± 0.02	5.10
23	5	60	4	150	376.52 ± 1.31	363.95	24.55 ± 0.64	23.07	1.51 ± 0.05	1.39	2.75 ± 0.05	2.45	1.72 ± 0.01	1.78
24	5	120	4	150	379.01 ± 0.37	378.56	22.28 ± 0.1	23.96	1.16 ± 0.05	1.22	2.44 ± 0.05	2.08	1.57 ± 0.02	1.74
25	5	90	4	100	513.43 ± 1.05	534.97	34.80 ± 0.42	33.88	1.86 ± 0.11	1.44	3.75 ± 0.03	2.98	2.64 ± 0.06	2.58
26	5	90	4	100	520.57 ± 0.66	534.97	35.55 ± 0.08	33.88	1.17 ± 0.06	1.44	3.24 ± 0.07	2.98	2.52 ± 0.04	2.58
27	5	90	4	100	570.92 ± 1.70	534.97	31.29 ± 0.08	33.88	1.29 ± 0.09	1.44	1.96 ± 0.02	2.98	2.59 ± 0.02	2.58

^1^ Mass concentration (m/V) of Brij S20 surfactant. ^2^ Solvent-to-material ratio. ^3^ Total phenolic content. ^4^ The mean values ± standard deviation (*n* = 3).

**Table 2:** ANOVA analysis and statistical parameters of the response surface models.

	TPC (mg GAE/L Extract)				DPPH Radical Inhibition (%)				Gallic Acid Concentration (mg/L)				Catechin Concentration (mg/L)				Quercetin Concentration (mg/L)			
Source	Contribution	Regression Coefficient	F-Value	*p*-Value	Contribution	Regression Coefficient	F-Value	*p*-Value	Contribution	Regression Coefficient	F-Value	*p*-Value	Contribution	Regression Coefficient	F-Value	*p*-Value	Contribution	Regression Coefficient	F-Value	*p*-Value
Intercept		1170				37.3				3.28				13.39				8.54		
Model	99.53%		182.32	<0.0001	98.78%		69.37	<0.0001	91.15%		8.83	<0.0001	94.15%		13.80	<0.0001	98.95%		80.71	<0.0001
Linear	89.77%		575.51	<0.0001	85.99%		211.38	<0.0001	61.19%		20.75	<0.0001	78.19%		40.10	<0.0001	87.46%		249.69	<0.0001
*X*_1_	0.37%	74.7	9.44	0.0106	0.40%	−1.69	3.92	0.0714	0.76%	−0.083	1.03	0.3304	1.16%	−0.265	2.38	0.1486	0.24%	−0.024	2.70	0.1264
*X*_2_	0.14%	3.66	3.64	0.0813	0.39%	0.012	3.83	0.0742	0.10%	−0.0262	0.14	0.7152	0.00%	0.005	0.01	0.9263	0.12%	0.0081	1.38	0.2632
*X*_3_	0.65%	4.0	16.71	0.0024	7.67%	15.9	75.38	<0.0001	5.51%	1.405	7.48	0.0186	1.36%	−0.396	2.78	0.1216	0.01%	−0.018	0.07	0.7980
*X*_4_	88.60%	−14.72	2272.26	<0.0001	77.54%	−0.473	762.38	<0.0001	54.81%	−0.0519	74.36	<0.0001	75.66%	−0.1314	155.24	<0.0001	87.10%	−0.09605	994.62	<0.0001
Square	9.47%		60.73	<0.0001	11.62%		28.55	<0.0001	28.56%		9.69	0.0015	15.66%		8.03	0.0023	11.13%		31.78	<0.0001
*X*_1_ *·X*_1_	1.35%	−5.4	6.33	0.0273	0.69%	0.52	5.62	0.0353	0.11%	−0.0021	0.00	0.9563	0.99%	−0.0057	0.01	0.9268	0.83%	−0.0113	0.35	0.5653
*X*_2_ *·X*_2_	0.79%	−0.00505	0.28	0.6060	0.26%	0.001185	1.48	0.2486	1.52%	0.000226	1.79	0.2063	0.94%	0.000045	0.03	0.8700	0.65%	0.000014	0.03	0.8694
*X*_3_ *·X*_3_	0.49%	2.28	1.13	0.3102	7.71%	−1.407	41.09	<0.0001	17.68%	−0.1307	11.81	0.0054	0.72%	0.0347	0.33	0.5764	0.78%	0.0102	0.29	0.6021
*X*_4_ *·X*_4_	6.84%	0.04548	175.33	<0.0001	2.95%	0.001893	29.05	<0.0001	9.25%	0.000216	12.55	0.0042	13.01%	0.000499	26.69	<0.0001	8.87%	0.000307	101.35	<0.0001
2-Way Interaction	0.29%		1.25	0.3475	1.17%		1.92	0.1593	1.41%		0.32	0.9154	0.30%		0.10	0.9943	0.36%		0.68	0.6692
*X*_1_ *·X*_2_	0.01%	−0.101	0.37	0.5532	0.06%	−0.0132	0.61	0.4503	0.03%	−0.0006	0.04	0.8408	0.01%	0.00063	0.02	0.8942	0.02%	0.00063	0.19	0.6743
*X*_1_ *·X*_3_	0.01%	−1.03	0.17	0.6853	0.07%	0.207	0.67	0.4312	0.00%	−0.0006	0.00	0.9892	0.06%	0.0245	0.12	0.7316	0.00%	0.0048	0.05	0.8302
*X*_1_ *·X*_4_	0.00%	0.0126	0.02	0.9017	0.66%	−0.0258	6.46	0.0263	0.29%	0.0011	0.39	0.5435	0.01%	0.00043	0.02	0.8803	0.12%	0.001021	1.35	0.2684
*X*_2_ *·X*_3_	0.24%	−0.41	6.16	0.0294	0.13%	−0.0187	1.23	0.2895	0.02%	−0.00051	0.03	0.8642	0.04%	−0.0014	0.09	0.7682	0.11%	−0.00168	1.31	0.2743
*X*_2_ *·X*_4_	0.01%	−0.00241	0.13	0.7226	0.05%	−0.000465	0.47	0.5043	0.36%	−0.000082	0.49	0.4992	0.18%	−0.000112	0.36	0.5584	0.07%	−0.000051	0.77	0.3972
*X*_3_ *·X*_4_	0.03%	0.0812	0.67	0.4292	0.21%	−0.0146	2.06	0.1762	0.70%	−0.00172	0.96	0.3483	0.00%	−0.00013	0.00	0.9643	0.04%	0.000566	0.41	0.5321
Error	0.47%				1.22%				8.85%				5.85%				1.05%			
Lack-of-Fit	0.27%		0.28	0.9324	0.96%		0.75	0.6923	7.21%		0.88	0.6417	3.19%		0.24	0.9521	1.03%		9.84	0.0964
Pure Error	0.19%				0.26%				1.64%				2.66%				0.02%			
Total	100.00%				100.00%				100.00%				100.00%				100.00%			

**Table 3:** Adequacy of the models tested.

Source	R^2^ (%)	Adjusted R^2^ (%)	Predicted R^2^ (%)
TPC (mg GAE/L extract)	99.53	98.99	97.99
DPPH radical inhibition (%)	98.78	97.36	93.87
Gallic acid concentration (mg/L)	91.15	80.84	54.81
Catechin concentration (mg/L)	94.15	87.33	75.64
Quercetin concentration (mg/L)	98.95	97.72	94.02

**Table 4:** Predicted values under optimum conditions.

Response	Predicted Values	95% CI	95% PI
TPC (mg GAE/L extract)	898.1	(849.3, 946.8)	(832.9, 963.2)
DPPH radical inhibition (%)	56.95	(51.97, 61.93)	(50.29, 63.61)
Gallic acid concentration (mg/L)	3.47	(2.605, 4.331)	(2.315, 4.622)
Catechin concentration (mg/L)	6.65	(5.277, 8.016)	(4.815, 8.477)
Quercetin concentration (mg/L)	5.03	(4.599, 5.462)	(4.453, 5.608)

**Table 5:** Total phenolic content and DPPH radical inhibition of grape pomace extracts of different varieties.

	TPC (mg/L)	DPPH (%)
*Cabernet Franc*	968.50 ± 37.06	61.41 ± 7.13
*Cabernet Sauvignon*	438.15 ± 2.73	25.86 ± 3.33
*Probus*	727.34 ± 41.47	45.64 ± 3.34
*Prokupac*	818.73 ± 16.57	57.17 ± 0.16
*Merlot*	685.38 ± 27.41	49.56 ± 1.69

**Table 6:** Concentrations of individual phenolic compounds in grape pomace extracts of different varieties.

	Concentration of Individual Phenolics (mg/L)								
	Gallic Acid	Vanillic Acid	Catechin	Rutin	Quercetin	Kaempferol	Naringenin	Hesperetin	Resveratrol
*Cabernet Franc*	5.10 ± 0.05	0.65 ± 0.01	10.62 ± 0.79	1.28 ± 0.18	6.04 ± 0.10	1.03 ± 0.05	0.16 ± 0.03	n.d.	0.16 ± 0.06
*Cabernet Sauvignon*	1.19 ± 0.08	0.93 ± 0.06	1.35 ± 0.12	5.26 ± 0.21	2.55 ± 0.12	0.63 ± 0.09	0.16 ± 0.05	n.d.	0.07 ± 0.001
*Probus*	1.98 ± 0.08	1.00 ± 0.03	4.20 ± 0.08	4.92 ± 0.15	3.21 ± 0.09	0.53 ± 0.08	0.15 ± 0.02	n.d.	0.20 ± 0.02
*Prokupac*	6.71 ± 0.01	0.86 ± 0.001	12.23 ± 0.11	3.17 ± 0.20	1.40 ± 0.05	0.49 ± 0.05	0.19 ± 0.03	n.d.	0.11 ± 0.02
*Merlot*	3.32 ± 0.11	1.26 ± 0.03	8.12 ± 0.34	4.37 ± 0.15	3.24 ± 0.10	0.59 ± 0.02	n.d.	0.27 ± 0.07	0.08 ± 0.01

n.d.—not detected.

**Table 7:** Levels of independent variables for the experimental design.

Independent Variable	Code Units	Coded Variable Levels		
		−1	0	+1
C (Brij S20) (%) ^1^	*X*_1_	3	5	7
Time (min)	*X*_2_	60	90	120
pH	*X*_3_	2	4	6
SM ratio (mL/g) ^2^	*X*_4_	50	100	150

^1^ Mass concentration (m/V) of Brij S20. ^2^ Solvent-to-material ratio.

## Data Availability

Data are contained within the article or Supplementary Material.

## Supplementary Materials

The following supporting information can be downloaded at https://www.mdpi.com/article/10.3390/ijms26052072/s1.
