# Supplementary figures and images for: Optimization of Surfactant-Mediated Green Extraction of Phenolic Compounds from Grape Pomace Using Response Surface Methodology
Source: Int J Mol Sci. 2025 Feb 27;26(5):2072. doi: 10.3390/ijms26052072 (PMC11900315; doi:10.3390/ijms26052072)

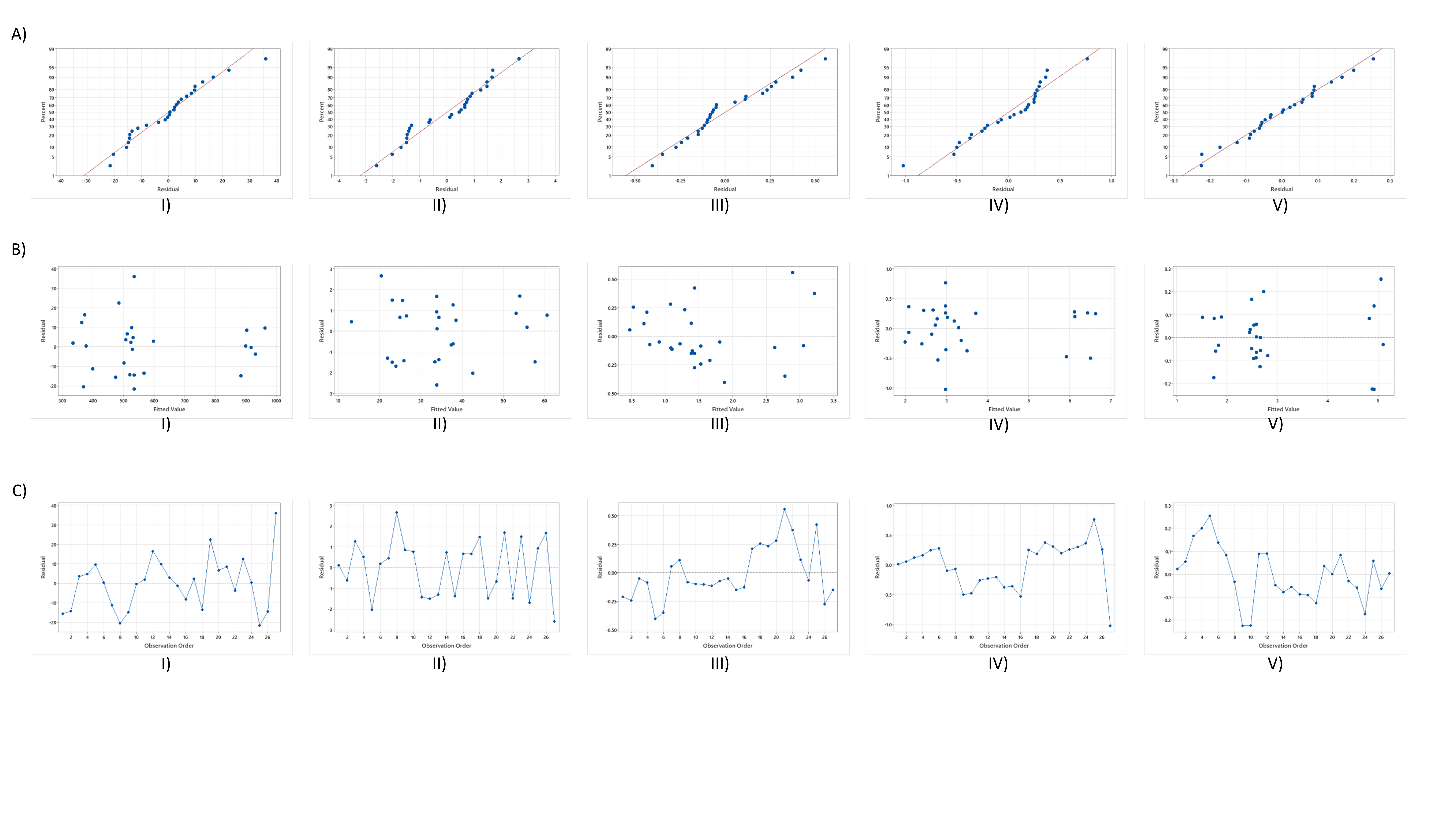

Supplement: Supplementary file 1 [file ijms-26-02072-s001.zip › ijms-3486439-supplementary.tif]
